# A case of atypical vivax malaria with a global review of reports on myriads of morpho-variations in parasitized red blood cells

**DOI:** 10.1099/acmi.0.000461.v3

**Published:** 2023-04-25

**Authors:** Debasish Biswal, Bijay Ranjan Mirdha

**Affiliations:** ^1^​ Department of Microbiology, All India Institute of Medical Sciences, New Delhi, India

**Keywords:** vivax malaria, hypovolaemia, morphological variations, microgametocytes, PCR assay

## Abstract

*Plasmodium vivax*, one of the major species associated with human malaria, continues to be a major public health problem in many parts of the world. Numerous studies related to vivax malaria have described quantitative haematological findings (level of haemoglobin, thrombocytopaenia, haematocrit values), but diverse morphological changes of parasite forms within infected red blood cells (iRBCs) have been mentioned only in few studies. Here we report a case of a 13-year-old boy who presented with fever, significant low platelet counts and hypovolaemia that created a diagnostic dilemma. Detection of microgametocytes by microscopic examinations, further confirmed by multiplex nested PCR assays and response to anti-malarials, helped to make the diagnosis. We present an atypical case of vivax malaria with a review of morpho-variations of iRBCs and have summarized the characteristics that aid in creating increased awareness among laboratory health professionals and public health workers.

## Data Summary

No supporting data, software or code was generated or re-used.

## Introduction

In geographical areas with *Plasmodium falciparum* and *Plasmodium vivax* co-endemicity, vivax malaria is relatively more widely distributed, causing singnificant morbidity and mortality [1]. In the 1940s, writing in a classical textbook of modern malariology, Kitchen explicitly described *P. vivax* as intrinsically benign, but such a description may no longer be viewed as a paradigm of vivax malaria [2]. *P. vivax* infects reticulocytes and young red blood cell (RBCs) [3] and rarely exceeds 2 % of circulating RBCs; with high parasite burden not being a usual feature, this may potentially lead to severe manifestations such as renal failure and cerebral malaria [4]. Classical clinical presentations of human malaria are present in 50–70 % of patients who may present with unusual features with or without complications. A systematic review of 26 studies of *P. vivax* malaria from eight different countries revealed an association of severe malaria, severe anaemia, acute respiratory distress syndrome (ARDS) and cerebral malaria [5]. Severe forms of *P. vivax* malaria can be attributed to long duration of the hepatic stage that allows the parasite to remain in the host even after interruption of transmission and successful treatment of the primary infection [6]. Furthermore, the continued presence of parasites seems to be sufficient to infect and destroy most of the reticulocytes, thereby delaying the timely replenishment of the erythrocyte population and leading to severe manifestations [7].

Various morphological changes in infected RBCs (iRBCs) due to *P. vivax* observed during microscopic examination of stained peripheral blood smears include (i) crisis forms, (ii) pseudo-parthenogenesis forms, (iii) equatorial trophozoites, (iv) phagocytosed parasites, (v) malaria pigment-containing phagocytes, (vi) microgametocytes with or without ex-flagellation, (vii) multiple invasions of iRBCs and (viii) macrogametocytes. Crisis forms appear as blackish-brown pigmented and are characterized by an extra-erythrocytic bluish amoeboid structure of the parasites. These are disintegrating forms of parasites and have been reported from India, often in co-infections with lymphatic filariasis [8]. The underlying reasons are not clear but it is possibly due to exaggerated host immunological responses of filarial co-infections [8].

‘Pseudo-parthenogenesis forms’ in *P. vivax* malaria were first described by Garnham *et al.* in 1966 where the simultaneous presence of both asexual and sexual forms were detected in the same iRBC [9, 10]. These forms have been reported from French nationals [10, 11]. In equatorial forms, the trophozoite is found aligned along the centre of iRBCs, as reported by Mazars *et al.* in 1997 [11].

Intra-leucocytic malaria pigment was first observed by Meckel in viscera and blood of patients who died of pernicious fever [12]. Such findings were subsequently reported by Lawrence *et al.* in 1986 [13] using polarized light and by Jamjoom *et al.* in 1983 using dark-field microscopy [14]. Malaria pigment, an end-product of haemoglobin digestion, has been observed in the peripheral blood of patients with severe malaria, and the high percentage of neutrophils containing malarial pigment reflects acute hyper-parasitaemia.

Phagocytosed malaria parasites have been reported in patients with extremely acute pernicious malaria. Almost a century ago, Golgi depicted circulating leucocytes containing malarial parasites either well preserved or in various stages of disintegration [15].

More commonly reported variations are microgametocytes with or without ex-flagellation. Such events occur often in definitive hosts but are rarely seen in peripheral blood smears of intermediate hosts. A heat-stable molecule derived from mosquito head and gut, namely mosquito ex-flagellation factor (MEF), is believed to be vital in inducing *in vitro* ex-flagellation [16]. During ex-flagellation, eight thin, long flagella measuring approximately 10–15 μm in length extend from the membrane, and the nuclear material traverses down each flagellum. These flagellated microgametes are actively motile, seeking to fertilize macrogametocytes [17]. This form was initially reported by MacCallum in 1897 in a patient with *P. falciparum* malaria [18]. At the time of writing, only 16 cases of ex-flagellated microgametocytes of *Plasmodia* species have been reported [19]. The occurrence of ex-flagellated microgametocytes in vivax malaria has also been reported [17, 20–24]. With detailed examination, ex-flagellated forms of *Plasmodia* occur as thin filamentous structures with an oval-shaped dark blue nucleus [16, 19, 20, 25]. In contrast, macrogametocytes are extra-erythrocytic, enlarged and elongated, measuring around 2.5–3.5 times that of the RBC diameter. These forms have been reported by Hahm *et al*. [26]. Multiple invasions of RBCs, predominantly considered as a feature of *P. falciparum* malaria, have also been reported in *P. vivax*. As many as five rings in a single iRBC in a neonate just 15 h after birth [27] and single, double and triple infections in 250 different infected RBCs have also been reported [28].

## Case presentation

A 13-year-old boy was referred to our hospital with complaints of high-grade fever, non-bilious, non-bloody projectile vomiting, light headedness, generalized weakness, reduced appetite and passing black-coloured urine. Prior to his admission, the child was febrile (40 °C) with blood pressure of 110/40 mmHg (considered to be low as per guidelines of the National Institute of Health [29]), along with thrombocytopaenia (28 000 mm^–3^ blood; normal: 1.5–4.5 mm^–3^ blood), low haematological parameters of haemoglobin (12.3 g dl^−1^; normal: 10–15.5 g dl^−1^), haematocrit (35.8 %; normal: 35–44 %), mean corpuscular volume (66.1 fl; normal: 80–95 fl) and mean corpuscular haemoglobin (22.7 pg; normal: 32–36 pg). Blood urea (62 mg dl^−1^; normal: 5–18 mg dl^−1^) and random blood sugar (204 mg dl^−1^; normal: 70–140 mg dl^−1^) levels were significantly raised. Peripheral blood smear examination was positive for *P. vivax*. Other tests such as Dengue (Non-structural protein 1) NS1, scrub typhus card tests, Widal test and COVID 19 reverse transcriptase (RT)-PCR were negative. Despite thrombocytopaenia, the child had no bleeding manifestations and was administered a first dose of intravenous artesunate and intravenous fluids.

**Fig. 1.**
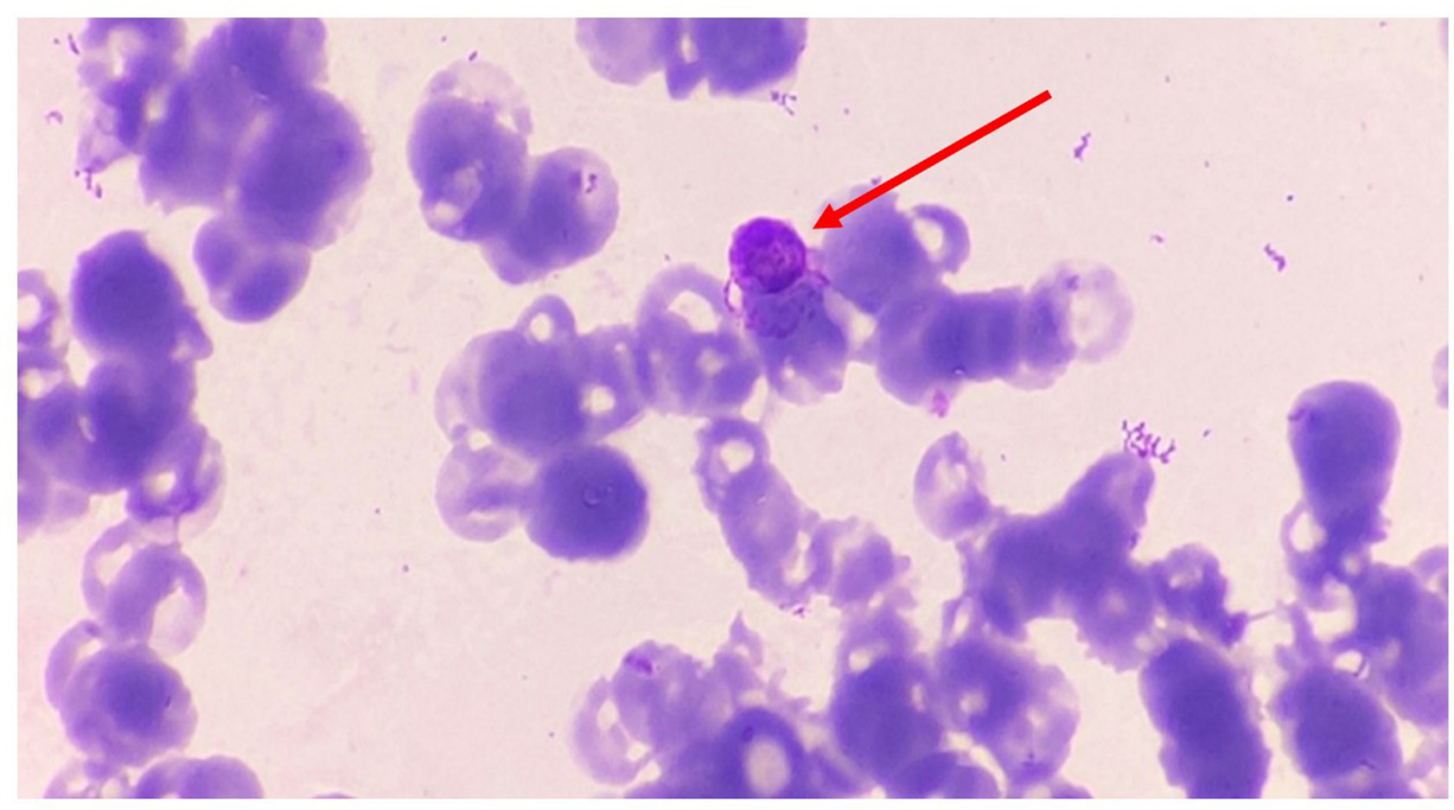
Microgametocyte 6.4 μm in diameter observed with pigments (×1000 magnification; Giemsa stain – red arrow).

**Fig. 2.**
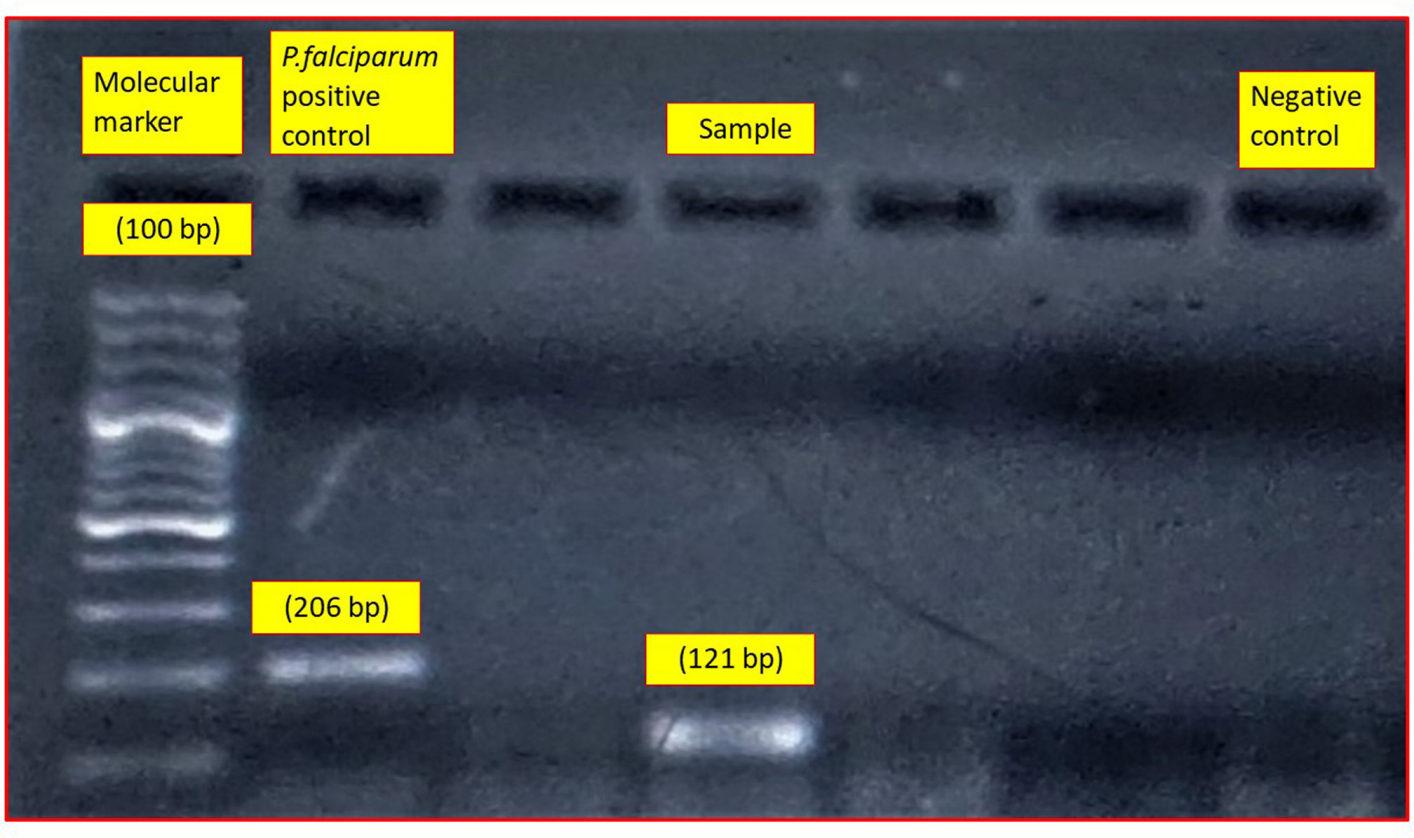
Identification of *Plasmodium* species by a species-specific nested PCR assay. Lane 1, 100 bp DNA ladder; Lane 2, positive control of *Plasmodium falciparum* (206 bp); Lane 4, sample positive for *Plasmodium vivax* (121 bp); Lane 7, negative control.

With the impending hypovolaemia, and persistent hypotension with an episode of loss of consciousness for 20 min, the child was referred to our hospital. He was afebrile, hypotensive (88/36 mmHg), with high pulse rate (110 bpm) and facial swelling. There was reduction in urine output of around 150 ml per day, but the oxygen saturation (S*p*O_2_) was well maintained. Liver was enlarged up to 2 cm below the right costal margin. Other systemic examinations were within normal limits. Upon laboratory investigations, his haemoglobin level was 11 g dl^−1^, with a low haematocrit (34.2 %), mean corpuscular volume (71 fl) and mean corpuscular haemoglobin (22.9 pg) values with platelet count of 36 000 mm^–3^ blood. Serum direct bilirubin and liver enzymes were elevated. Giemsa-stained peripheral blood smears revealed normal sized erythrocytes with microgametocytes containing malarial pigments (Fig. 1). Quantitative buffy coat (QBC) examination detected gametocytes of non-*falciparum* species. Blood sample was subjected to a multiplex nested PCR assay using published primers (Table 1), and an amplified product of 121 bp corresponding with *P. vivax* was detected by 1.5 % agarose gel electrophoresis using a molecular ladder of 100 bp (Thermo Scientific Gene Ruler 100 bp ladder) (Fig. 2). Buffer used for the agarose gel cast and running buffer in the gel electrophoresis tank was 10× Tris boric acid EDTA (TBE). The child was treated with injectable inotropes, intravenous fluids, oxygenation, the remaining two doses of artesunate and injectable antibiotics (ceftriaxone and vancomycin). Antibiotics were discontinued after malaria was reported. A repeat complete haemogram showed a fall of platelets to 19 000 mm^–3^ blood after 7 h of treatment, but the child became stable after 22 h with the same regimen and was discharged with advice to continue artemether-lumefantrine combination without any other drugs and/or antibiotics.

**Table 1.** Primer sequences used for malaria detection (target gene: SSU 18S rRNA of circum-sporozoite protein, CSP)

*Plasmodium* genus nest 1 (1.6–1.7 kb)	rPLU1	5′ TCAAAGATTAAGCCATGCAAGTGA 3′
	rPLU5	5′ CCTGTTGTTGCCTTAAACTTC 3′
*Plasmodium* genus nest 2 (235 bp)	rPLU3	5′ TTTTTATAAGGATAACTACGGAAAAGCTGT 3′
	rPLU4	5′ TACCCGTCATAGCCATGTTAGGCCAATACC 3′
*P. falciparum* (206 bp)	rFal1	5′ TTAAACTGGTTTGGGAAAACCAAATATATT 3′
	rFal2	5′ ACACAATGAACTCAATCATGACTACCCGTC 3′
*P. vivax* (121 bp)	rVIV1	5′ CGCTTCTAGCTTAATCCACATAACTGATAC 3’
	rVIV2	5′ ACTTCCAAGCCGAAGCAAAGAAAGTCCTTA 3′
*P. ovale* (226 bp)	rOVA1	5′ ATCTCTTTTGCTATTTTTTAGTATTGGAGA 3′
	rOVA2	5′ GGAAAAGGACACATTAATTGTATCCTAGTG 3′
*P. malariae* (145 bp)	rMAL1	5′ ATAACATAGTTGTACGTTAAGAATAACCGC 3′
	rMAL2	5′ AAAATTCCCATGCATAAAAAATTATACAAA 3’
*P. knowlesi* (200 bp)	Pkr140(F)	5′ CAGAGATCCGTTCTCATGATTTCCATGG3′
	Pkr140(R)	5′ CTRAACACCTCATGTCGTGGTAG3′

## Discussion

Severe and complicated *P. vivax* infection can manifest clinically as that of *P. falcipraum* infection and has been previously reported in the literature. A case series [30] describes three cases of severe *P. vivax* malaria simulating that of *P. falciparum* infection. In each case, blood pressure was 80/50 mmHg, haemoglobin 9 g dl^−1^, platelets 70 000 mm^–3^ blood, and both chloroquine and artesunate was continued along with other supportive medications, which resulted in improvement of vital signs over the next 48 h in these patients. In another case, severe *P. vivax* malaria mimicked sepsis in a neonate, with low hemoglobion (4.4 g dl^−1^), hematocrit (13.1 %) and platelet count (95 000 mm^–3^ blood). The child improved with administration of injectable artesunate [31]. Two other cases of severe vivax malaria with similar conditions have also been reported [32]. Such events may plausibly be explained by some *P. vivax* proteins such as Pv RBP1 and Rp RBP2 that are thought to restrict *P. vivax* to reticulocytes [33].

In recent years, severe forms of malaria due to *P. vivax* have been increasingly reported, suggesting a diversity of *P. vivax* isolates in different parts of the world [34]. The growing number of *P. vivax* cases in duffy antigen receptor for chemokines (DARC)-negative individulas further adds to the changing paradigm [35, 36]. In this context, next generation sequencing (NGS) of four geographically distinct *P. vivax* isolates supported the distinctiveness of transmissibility and spectre of *P. vivax* related manifestations [37]. *P. vivax* infection can be identified with the help of latency, patency and relapse. The periodicity of *P. vivax* varies systematically in different regions, indicating adaptations in the variability of iRBCs. Presentations mimicking dengue in the present case with significant thrombocytopaenia and without haemorrhagic manifestations is another facet, although is not unusual. Malaria and dengue co-infections have been reported most frequently from the Indian subcontinent and often cause misdiagnosis and/or misinterpretation of mono-infections [38]. Variations in clinical manifestations due to *P. vivax* malaria indicate interactions among host, parasite and external factors, of which parasite virulence seems to play a major role [39, 40]. Anti-malarial drug resistance, invasion of Duffy-negative erythrocytes, production of anti-erythrocyte antibodies and above all *P. vivax* polymorphisms altering cytokine production are other important factors [41]. Peripheral blood smear examination is the mainstay of malaria diagnosis, albeit with limitations. A myriad of morphological variations as described in peripheral blood smears of patients infected with *P. vivax* shows its distinctiveness with underlying pathogenetic mechanisms. The recognition of such morphological changes of iRBCs is important to avoid diagnostic dilemmas.

## Conclusion

What makes our case interesting is that our patient had a very low thrombocytopaenia with no bleeding diathesis but with severe hypovolaemia without manifesting with any features of shock or sepsis. In addition, a collective investigative algorithm was able to conclusively achieve a diagnosis.
